# Convolutional neural network for gesture recognition human-computer interaction system design

**DOI:** 10.1371/journal.pone.0311941

**Published:** 2025-02-19

**Authors:** Peixin Niu

**Affiliations:** Design Department, Taiyuan Normal University, Jinzhong, Shanxi, China; Manipal Institute of Technology, Manipal Academy of Higher Education, INDIA

## Abstract

Gesture interaction applications have garnered significant attention from researchers in the field of human-computer interaction due to their inherent convenience and intuitiveness. Addressing the challenge posed by the insufficient feature extraction capability of existing network models, which hampers gesture recognition accuracy and increases model inference time, this paper introduces a novel gesture recognition algorithm based on an enhanced MobileNet network. This innovative design incorporates a multi-scale convolutional module to extract underlying features, thereby augmenting the network’s feature extraction capabilities. Moreover, the utilization of an exponential linear unit (ELU) activation function enhances the capture of comprehensive negative feature information. Empirical findings demonstrate that our approach surpasses the accuracy achieved by most lightweight network models on publicly available datasets, all while maintaining real-time gesture interaction capabilities. The accuracy of the proposed model in this paper attains 92.55% and 88.41% on the NUS-II and Creative Senz3D datasets, respectively, and achieves an impressive 98.26% on the ASL-M dataset.

## 1. Introduction

Gesture expression serves as an alternative means of communication distinct from spoken language, possessing convenient and intuitive characteristics. It aids individuals in effectively conveying ideas and facilitates the transmission of emotions in everyday interactions. Gesture recognition plays a pivotal role in bridging communication and connection between users and machines, emerging as an essential interaction mode and key technology within the realm of human-computer interaction. Its widespread application spans various fields, including augmented reality, driverless driving, and other domains of human-computer interaction [1]. Consequently, the study of gesture recognition is deemed both necessary and meaningful.

The exploration of gesture recognition emerged in the public domain during the 1980s, primarily facilitated by wearable sensor devices for acquiring gesture-related data. Among the diverse sensors employed, the data glove device stands out as the most prominent. To achieve comprehensive gesture recognition, the numerous sensors embedded in the data glove capture real-time spatial hand positions, finger curvature, and hand movement trajectories [2]. The utilization of sensor devices in gesture identification offers advantages such as accurate detection and high stability, leading to a significant surge in the research and development of gesture recognition. Neuromorphic sensors encompass dynamic vision sensors, with distributed switch-based event cameras offering several advantages over traditional counterparts. These advantages include heightened time resolution, reduced power consumption, and diminished data redundancy. Consequently, there is an enhanced suitability for computer vision tasks such as gesture recognition. In a study referenced as [3], in their research on EMG-based gesture recognition, Fariman et al. compared the learning and recognition performance of ANFIS and ANN classification algorithms in terms of model training time, classification time and classification accuracy.in [4], the research on dynamic gesture recognition, a Convolutional Neural Network (CNN), combined with the pattern recognition model of FMMNN, is proposed to obtain a recognition rate equivalent to that of all the features in the classification experiment of six dynamic gestures. About 89.17%..in [5], Lin proposed TSM(Temporal Shift Module), which promotes information interaction between different time frames through channel shift operations, and improves the shortcomings of 2D CNN in time relationship modeling in dynamic gesture recognition. in [6], Wang proposed three sub-modules: spatio-temporal excitation module, channel excitation module and motion excitation module, and combined with channel shift operation to carry out spatio-temporal information interaction and motion information capture. in [7], Lee proposed an action feature network including action modules, which can be used at the end spatiotemporal information between adjacent frames is encoded in the end-to-end training network, thus obtaining orders in gesture datasets such as Jester people are satisfied with the recognition effect, the author in [8] employs the Kinect composite sensor device to capture depth images of gestures. A sequence binary image extraction scheme is proposed in this study to effectively minimize the interference of shadow effects on gesture recognition and extract key features from gesture depth images. In [9], the authors proposed a novel sensor-based continuous gesture recognition technique called Long Short-Term Memory (LSTM). This algorithm requires only a basic accelerometer and/or gyroscope. By inputting series of sensing data and adopting a many-to-many LSTM scheme, the algorithm generates an output path, enabling continuous gesture recognition. In [10], the authors propose a differential evolution technique with the objective of maximizing the weight of dynamic time distortions for multi-sensory gesture recognition. The primary goal is to develop a reliable gesture detection system customizable for various contexts, employing the Kinect sensor for verification and data collection as a multi-sensor. While the existing research on sensor-based gestures has reached a level of maturity, the substantial volume of data introduces complexity in computation and consumes hardware memory resources during data processing. Consequently, it becomes imperative to explore lightweight data processing techniques, and this serves as the driving force behind our current investigation.

Researchers have favored vision-based gesture recognition methods due to their portability and flexibility, leading to their gradual integration into daily life. Traditional approaches in vision-based gesture recognition involve initial segmentation of the gesture region. Subsequently, manual feature extraction methods, such as orientation gradient histogram and SURF, are employed. Finally, classifiers such as support vector machines and random forests are utilized to categorize the gesture and ascertain its semantics [11]. In [12], the authors employed color space detection to segment the hand image. Subsequently, color and edge features were extracted from the segmented image, and the gesture classification was accomplished using Support Vector Machine (SVM). This study introduced a novel method for gesture recognition, utilizing color and edge features in conjunction with SVM for effective gesture classification. Moreover, the authors proposed an application for gesture recognition aimed at overriding control car interfaces. In [13], a gesture recognition system was proposed. To enhance the gesture recognition rate, hand skin color detection was partially conducted in the YCbCr color space. The salient features of the hand were then used to extract feature points that facilitate recognition. In [14], effective human-computer interaction is accomplished through the tracking and continuous localization of hand regions using solely a webcam. The region of interest is identified within the captured image and classified for task-specific gestures. Following experimental validation, the proposed system demonstrated a remarkable recognition rate of 98.44% suitable for practical, real-time applications. In summary, conventional recognition methods necessitate the specific design of a feature extraction scheme. Relying on manual feature extraction methods introduces a dependency on researchers’ experience, resulting in substantial deviations in experimental outcomes and elevated research and development costs. Additionally, factors such as the gesture background and external environmental interference further necessitate optimization for the performance of gesture recognition algorithms.

A novel approach to gesture identification has arisen from the successful application of deep learning in the fields of image classification and segmentation. Gesture recognition methods based on deep learning, leveraging automatic feature extraction, demand less a priori knowledge from researchers and eliminate the need for intricate extraction procedures. This development has contributed to the incremental improvement of the accuracy challenge in gesture recognition [15, 16]. The authors customized two Convolutional Neural Network (CNN) models to identify 24 static gestures in American Sign Language (ASL), relying solely on the convolutional and fully connected layers. The test results illustrated improved performance of the model [17]. In a study by Barbhuiya et al. [18], an enhanced AlexNet network was employed for recognizing ASL gestures. Hamid et al. [19] conducted training on various gesture recognition tasks using AlexNet and GoogleNet networks. They proposed a recognition application based on embedded systems, achieving a classification accuracy of up to 99.6%.

With the growing popularity of natural gesture interaction and the deepening exploration of deep learning, considering the comprehensive factors of economic cost and timeliness, there is a noticeable increase in the demand for applying convolutional neural network (CNN) algorithms to mobile or embedded devices. Simultaneously, there is a rising need for stringent performance and recognition speed requirements for network models. Among these, MobileNet [20] stands out as a noteworthy example of a lightweight network, incorporating a distinctive design approach. Instead of employing conventional convolution with extensive parameters, it adopts depth-separable convolution with fewer parameters. This approach ensures smooth information exchange while significantly reducing the computational complexity of the network. Presently, traditional gesture recognition models outperform others in both recognition speed and accuracy. He et al. [21] suggested an ultra-high-speed recognition technique based on MobileNetV2 to enhance this performance. Zhang et al. [22] employed models such as MobileNetV2, ShuffleNet, and others to assess the efficacy of millimeter-wave radar-based gesture recognition for prospective industrial applications. Qiang et al. [23] utilized the SqueezeNet network to extract hand features, enhance computational efficiency, and achieve a high-performance gesture recognition algorithm. Ling et al. [24], building upon the MobileNet network, incorporated a hierarchical attention mechanism to extract more profound semantic information, thereby enhancing the generalization of gesture recognition.

Convolutional Neural Networks (CNNs) play several crucial roles in the design of gesture recognition systems for human-computer interaction [25]. As a subset of deep learning models, CNNs have demonstrated high effectiveness in analyzing and interpreting visual data, making them well-suited for recognizing and comprehending gestures. Various key roles of CNNs in gesture recognition systems will be discussed. In terms of feature extraction, CNNs excel at automatically extracting relevant features from raw image or video data. In gesture recognition, they can identify and capture distinctive patterns, edges, and shapes within the gestures. This feature extraction step is vital for discerning meaningful information in the input data. Regarding spatial hierarchies, CNNs are tailored to capture spatial hierarchies of features, a crucial aspect for recognizing gestures that often involve complex hand or body movements with spatial dependencies and hierarchical structures. CNNs can adeptly learn to identify and model these hierarchies. Gesture recognition systems must contend with variations in lighting conditions, background clutter, and diverse hand or body shapes and sizes among different users. Convolutional Neural Networks (CNNs) can achieve robustness against such variations by learning from extensive datasets encompassing diverse examples, rendering them well-suited for real-world applications. In terms of transfer learning, CNNs initially trained on large image datasets like ImageNet can serve as pre-trained models for gesture recognition. This approach enables developers to leverage existing CNN architectures, fine-tuning them on a smaller, gesture-specific dataset, thereby mitigating the need for an extensive labeled dataset [26–30]. Furthermore, many human-computer interaction systems necessitate real-time or near-real-time gesture recognition. CNNs can be optimized for efficient inference on hardware accelerators, making them suitable for applications like gesture-controlled gaming or user interfaces. Gesture recognition systems often integrate visual data with other sensors like depth sensors (e.g., Microsoft Kinect) or inertial sensors (e.g., accelerometers and gyroscopes). CNNs can collaborate with other deep learning models to fuse multi-modal data sources, enhancing the accuracy of gesture recognition. In continuous learning scenarios, CNNs can adapt and learn over time, which is valuable for systems dealing with the introduction of new gestures or the need to adapt to individual users’ gestures. Moreover, CNN-based gesture recognition enhances the user experience by enabling more intuitive interactions with computers, gaming consoles, or devices, reducing the reliance on physical controllers. Ultimately, for gesture classification, CNNs play a crucial role in the final step of categorizing recognized gestures into specific commands or actions, triggering appropriate responses in the human-computer interaction system.

The comparisons of existing literatures are shown in Table 1 below.

**Table 1:** Literature comparison table.

Title	Author	Summary	PubTime
Design and implementation of gesture recognition control system for unmanned vehicle based on MediaPipe	Xue Bintian; Shen Yubin	Combined with the characteristics of MediaPipe technology, a new gesture recognition control method of unmanned vehicle based on MediaPipe is proposed. The feasibility of the proposed scheme is verified by setting up a gesture recognition control platform for unmanned vehicle based on MediaPipe.	2024-03-18
Design of human-computer interaction system for Chinese speech recognition based on convolutional neural network	Han Xiangyang	In order to improve the recognition accuracy of Chinese speech recognition system, a human-computer interaction system for Chinese speech recognition based on convolutional neural network is proposed. The residual network and maxout function are integrated into the acoustic model of the system to improve the performance of the acoustic model. The results show that the optimized CTC-DCNN(maxout) model has better recognition performance.	2023-07-25
Design of interactive distance teaching system for electronic information based on multi-neural network	Wei Jiao; Xu Dan	In order to further improve the overall quality of distance teaching in China, a multi-neural network based human-computer interactive distance teaching system is proposed for electronic information courses. The fingertip trajectory is tracked through dynamic fingertip recognition, and the fingertip trajectory is planned and operated through static gesture recognition. The experimental results show that the proposed gesture recognition system can achieve accurate gesture type recognition with an average accuracy of more than 98%.	2023-02-25
Design of gesture visual recognition system for parent-child game consoles based on human-computer interaction	Lai Yanfang	This paper designs a gesture recognition system for parent-child game consoles, establishes a multi-layer perception model to recognize gesture contours, obtains induction recognition incentive function, generates activation signal, and realizes gesture recognition in parent-child game consoles. According to the experimental data, the accuracy of this recognition method is higher than other methods in static and dynamic recognition, which shows that the recognition effect of this method is better.	2022-07-25
Research and system design of two-hand gesture recognition based on RGB-D	Chang Zhilei	In this paper, depth information is introduced to estimate the key points of hand gestures in the left and right hand regions, and distance information is added to improve the accuracy of regression. A two-stage key point estimation algorithm is proposed to solve the influence of background and human body on the precision of gesture regression. Finally, the key points of prediction are matched by the template, and the result of hand gesture recognition is obtained by analyzing the bending state of the left and right hands.	2022-06-12
CSI cross-scene gesture recognition method based on 3D convolutional neural network	Wang Chi; Chang Jun	A CSI cross-scene gesture recognition method based on 3D convolutional neural network is proposed. The system extracts features unrelated to the scene and combines the 3D convolutional neural network learning model to realize cross-scene gesture recognition. In the experiment, network open data sets are used to verify the method, and the results show that the method recognizes 6 different gestures on average in known scenes The accuracy rate reached 86.50%, and the average recognition accuracy in unknown scenes reached 84.67%, which could realize cross-scene gesture recognition.	2021-04-21

In general, Convolutional Neural Networks (CNNs) stand as a foundational technology in the realm of gesture recognition, facilitating the creation of advanced and precise systems for diverse applications, including gaming, virtual reality, sign language translation, and beyond. Despite the notable achievements in achieving high accuracy rates in gesture recognition, there remains a gap in addressing considerations such as time efficiency, research costs, network computing efficiency, and application scenarios. This limitation hinders the broader promotion of related applications and underscores the need for further research in this domain.

According to the preceding analysis, the following are the primary contributions of this paper:

(1) Utilizing the MS-MobileNet network in conjunction with a skin color detection algorithm enables accurate segmentation of the hand area, effectively eliminating interference from the background environment.
(2) We incorporate a multi-scale convolution module to underscore the intrinsic features within image data, thereby augmenting feature extraction capabilities. Furthermore, the Exponential Linear Unit (ELU) activation function is utilized to maintain the integrity of information.
(3) The improved scheme is assessed through comparative experiments conducted on ASL-M, NUS-II, and Creative Senz3D datasets, enabling a comprehensive analysis of its strengths and weaknesses.

## 2. Basic theory

### 2.1 Gesture recognition dataset

**Fig 1:**
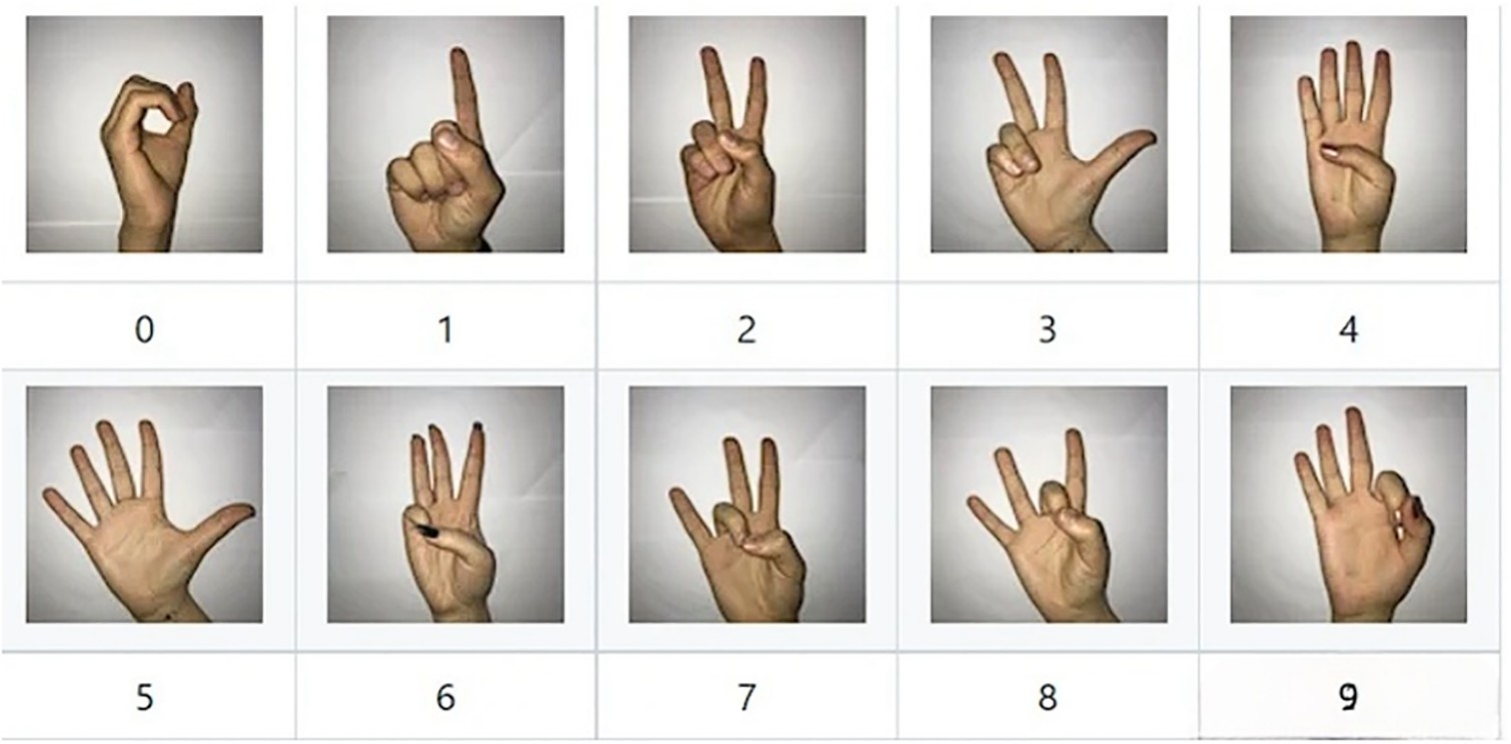
Map of selected datasets from ASL [26].

**Fig 2:**
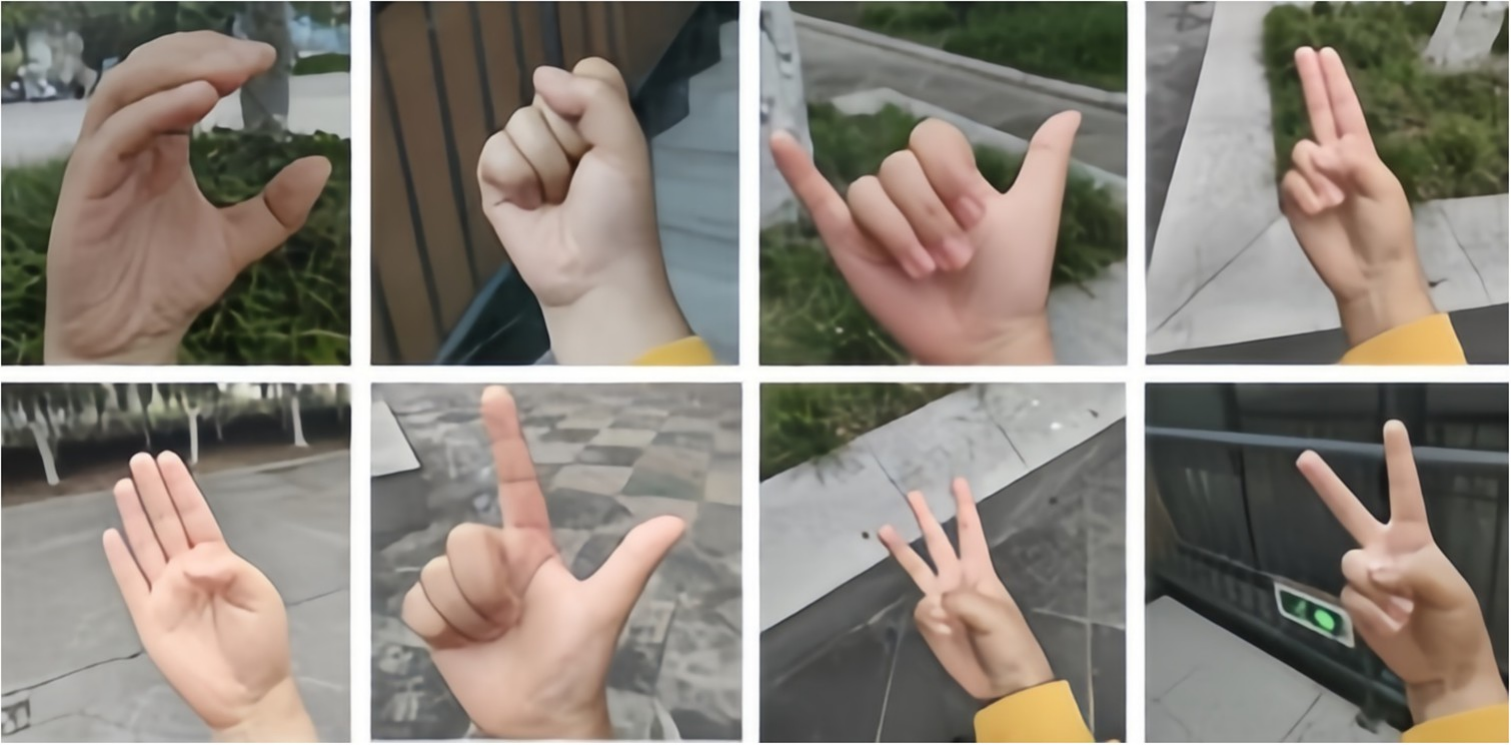
Self-constructed partial dataset maps.

The American Sign Language (ASL) Alphabet Sample dataset, designed to account for variations in background, light intensity, and skin color, serves as the origin of the ASL dataset. Derived from the ASL alphabet sample, this dataset encompasses diverse backgrounds, light intensities, hand shapes, skin tones, and other characteristics. The ASL collection comprises 29 types of gestures, including 26 gestures representing the letters A–Z, along with three additional forms for deletion, space, and none. Each gesture type encompasses 3,000 photographs, resulting in a total of 87,000 gesture images. Due to the ASL dataset being predominantly collected under specific conditions, lacking real sample data from application scenarios, this paper focuses on 24 gesture types (A-Z, excluding J and Z) for the ASL-M gesture recognition dataset. A self-built dataset, derived from ASL sign language gesture sample images, is incorporated, considering various conditions such as indoor and outdoor settings, different lighting, and backgrounds, to align with practical application scenarios. The selection includes 2000 samples for each gesture from the ASL data and 1200 samples for each gesture from the self-constructed data, summing up to 76,800 samples for gesture recognition research. Representative gesture data is depicted in Figs 1 and 2.

**Fig 3:**
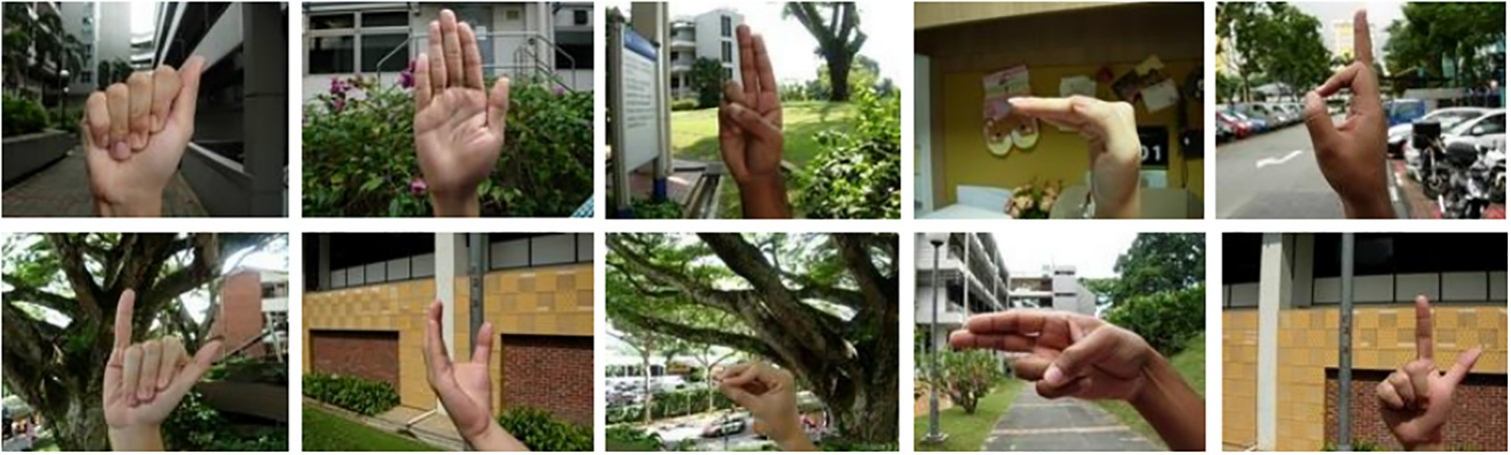
Sample diagram of a portion of the NUS-II dataset [27].

The NUS-II dataset is supplied by the National University of Singapore [27]. Forty participants, ranging in age from 22 to 56, were recruited for the dataset, encompassing individuals of various named ethnicities, diverse natural backgrounds, and varying hand sizes. Each participant was instructed to execute 10 gestures corresponding to the letters a-j in the alphabet, with each gesture repeated 5 times, resulting in a total of 2000 gestures. The additional category comprised 10 gestures containing noise, totaling 750 samples. Representative samples of these gestures are presented in Fig 3.

## 3. Gesture recognition based on MS-MobileNet network

### 3.1 Overall network structure

**Fig 4:**
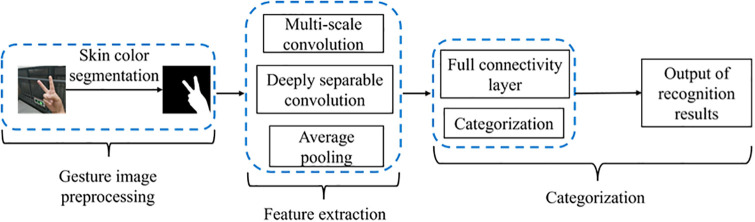
Gesture recognition flowchart.

Sign language serves as an auxiliary mode of communication, promoting seamless interactions among individuals and bridging the communication gap between the deaf community and the broader society. Furthermore, its utilization in the industrial sector holds the potential to contribute significantly to societal advancement. Within this framework, this study integrates skin color detection with the MS-MobileNet network to explore a hand gesture recognition algorithm. The process involves three crucial stages: gesture image preprocessing, feature extraction, and recognition classification. Refer to Fig 4 for an illustration of the algorithm’s workflow.

**Fig 5:**
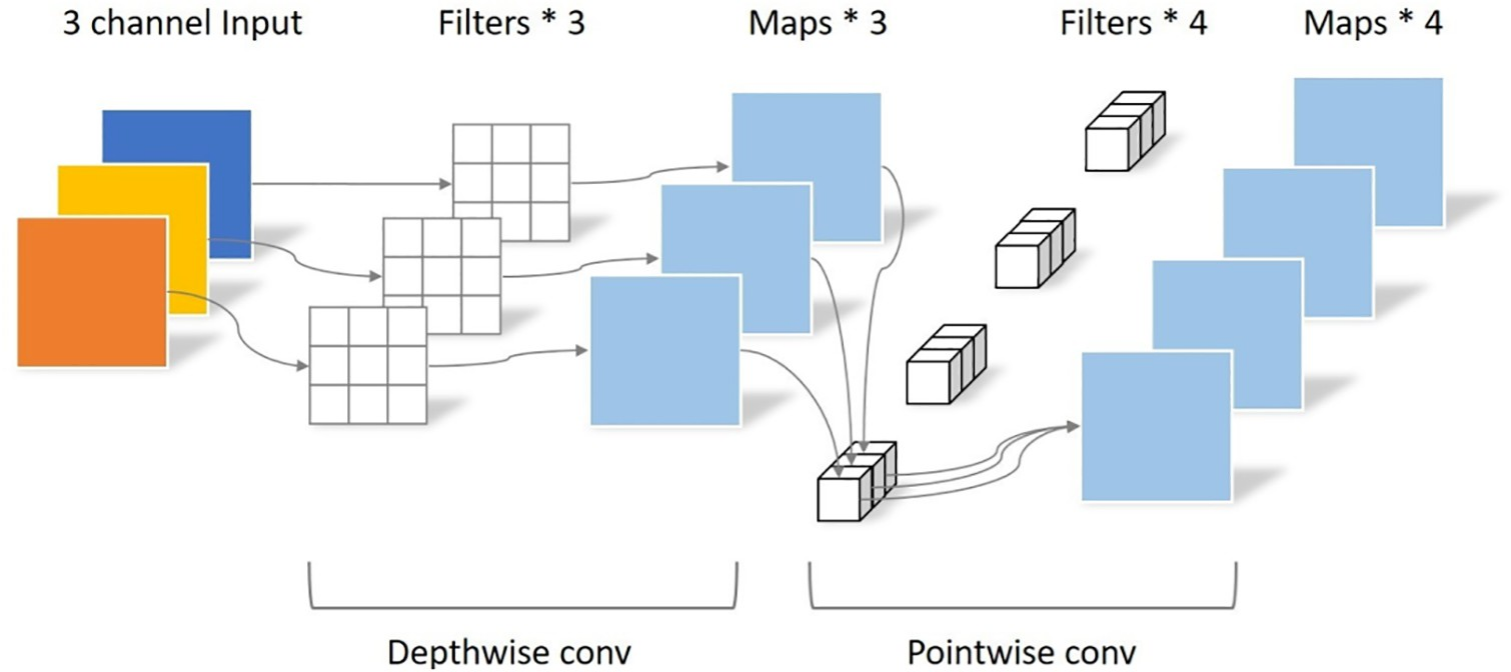
Multiscale convolutional framework.

Gesture image preprocessing is the first step in the gesture recognition process, which aims to enhance the information of the gesture region in the image while suppressing or eliminating background noise and interference. Feature extraction is a key step in gesture recognition, and its goal is to extract features from the preprocessed gesture image that can effectively distinguish different gestures. In the MS-MobileNet algorithm, a multi-scale convolution module is utilized to extract the underlying features, and the multi-scale convolution framework is shown in Fig 5 below. This step aims to enhance the feature extraction capability of the network to capture the subtle differences in the gesture images, and at the same time, combined with the lightweight MobileNet model suitable for the Web, the real-time and computational efficiency of the algorithm can be improved while ensuring the accuracy. Classification prediction is the final step of the gesture recognition process, which is based on the extracted gesture features, utilizes a classifier to classify the gestures, and outputs the final recognition results. In MS-MobileNet algorithm, by combining the trained classifiers, accurate and fast classification prediction can be performed on the input gesture images.

### 3.2 Gesture image preprocessing based on skin color detection

In the gesture recognition method, the camera collects gesture image information, which is then stored in the default RGB coding format. However, RGB-based color space coding is susceptible to variations in light intensity and exhibits limited generalization ability. In contrast, the YCbCr color space demonstrates superior resistance to light interference and exhibits improved clustering characteristics. The YCbCr color space (especially the YCbCr 4:2:0 or 4:2:2 formats commonly used in JPEG compression) is better suited for tasks such as skin color detection because it separates the brightness information (the Y component) from the chrominance information (the Cb and Cr components), making skin color detection more robust to changes in lighting. Furthermore, the conversion from the RGB color space to the YCbCr color space is linear, involving fewer computations and yielding a more effective segmentation effect. The conversion of YCnCr is expressed by Eq (1) [18]:

$$\left[\begin{array}{c} Y\\ Cb\\ Cr \end{array}\right]=\left[\begin{array}{ccc} 0.257 & 0.504 & 0.098\\ -0.148 & -0.291 & 0.439\\ 0.439 & -0.368 & -0.071 \end{array}\right]\left[\begin{array}{l} R\\ G\\ B \end{array}\right]+\left[\begin{array}{c} 16\\ 128\\ 128 \end{array}\right]$$ (1)

Where *Y* represents the luminance component, *Cb* and *Cr* represent the blue and red chromaticity offsets, respectively.

**Fig 6:**
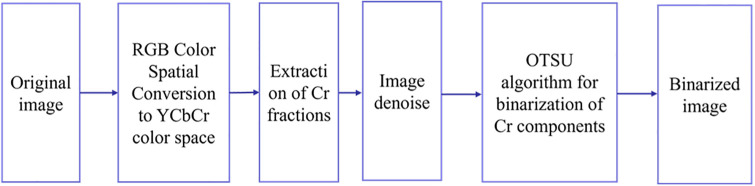
Flowchart of gesture skin color segmentation.

Following the conversion to the color space, the red component undergoes OTSU binarization [28] utilizing the Otsu algorithm. This approach leverages the natural characteristic of human skin color, which is a prominent feature in hand gestures. Employing the Otsu algorithm enhances resistance to factors like brightness and contrast, ensuring rapid computational speed. Importantly, it eliminates the need for manually setting thresholds, reducing the likelihood of errors and improving the accuracy of skin color detection. Consequently, this process effectively isolates the hand gesture from the image backgrounds, as illustrated in Fig 6.

### 3.3 Establishment of MS-MobileNet network

The advancement of intricate network models for image classification tasks faces constraints, including heightened latency, increased memory usage, and the requirement for sophisticated hardware infrastructure. To tackle these challenges and optimize the performance of complex network models, we propose a gesture recognition algorithm named MS-MobileNet. This algorithm is constructed upon the MobileNet network, with specific enhancements in convolution techniques and activation functions. The architecture of the network is depicted in the figure, where the MobileNet network acts as the foundational framework.

#### 3.3.1 Depth-separable convolution

**Fig 7:**
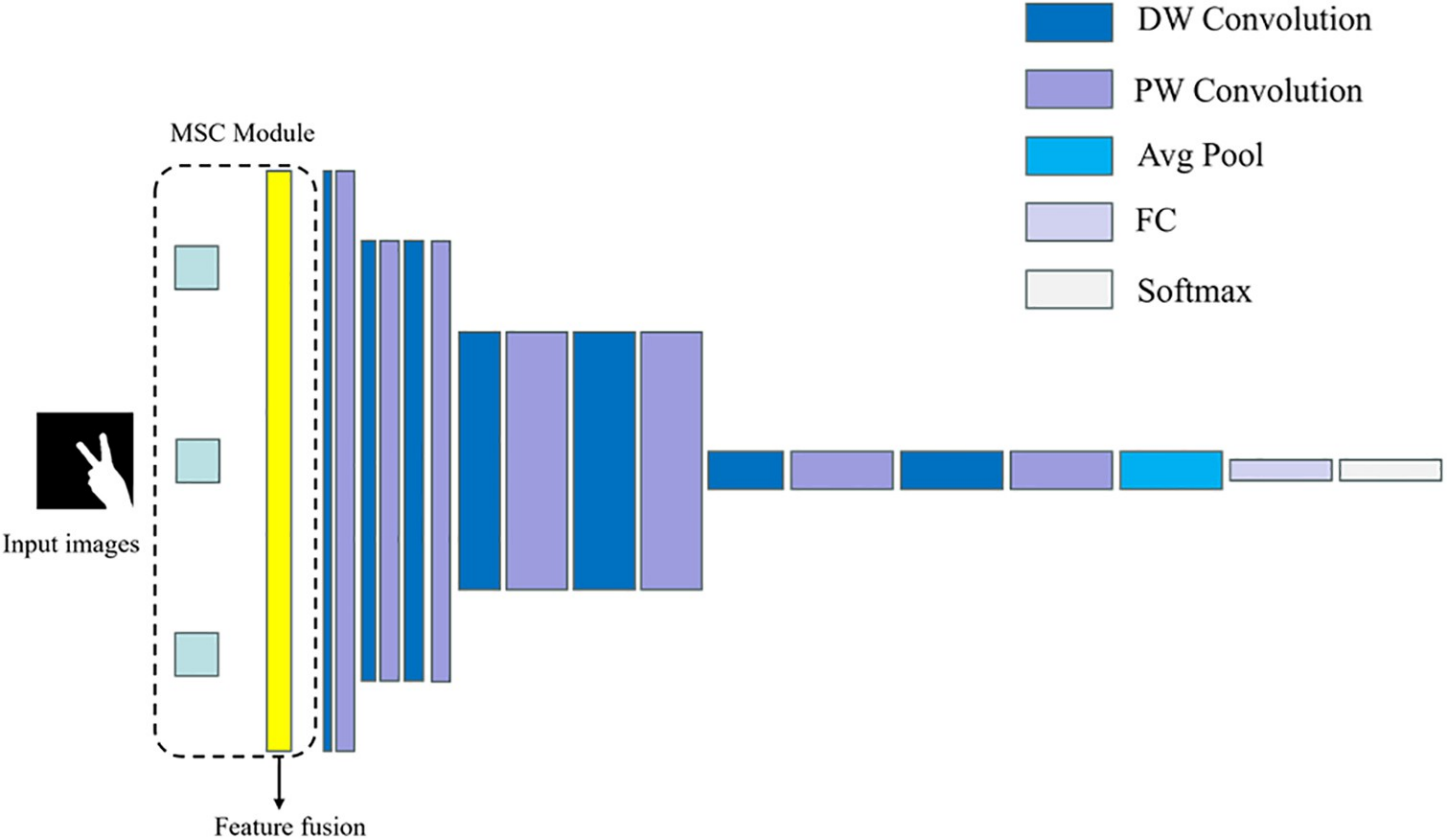
Standard convolutional map.

**Fig 8:**
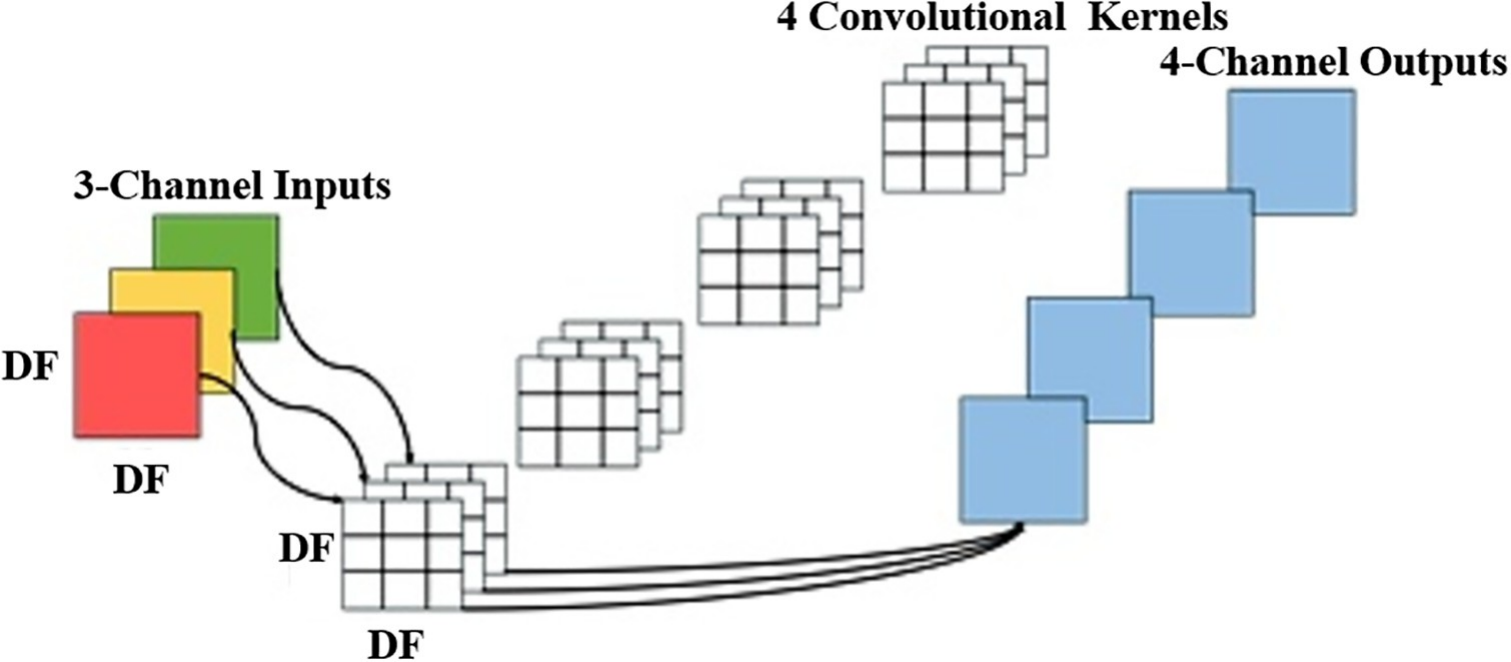
Deeply separable convolutional maps.

A unique type of convolution, called depth-separable convolution, distinguishes itself from conventional convolution by separating depth convolution from pointwise convolution. In this approach, deep convolution supervises the convolution operation, with each input channel aligned with a single convolution kernel, and the number of convolution kernels matched to the number of channels. On the other hand, point-by-point convolution manages feature fusion, essentially using standard 1x1 convolution kernels. The conventional convolution process is shown in Fig 7, while Fig 8 illustrates the deep separable convolution technique.

As shown in Fig 7, in the standard convolution process, the input feature dimension *F* is (*D*_*F*_, *D*_*F*_, *M*). The convolution operation is performed by using *N* standard convolution kernel *K* with dimension (*D*_*k*_, *D*_*k*_, *M*), Then the output feature dimension *G* size is (*D*_*G*_, *D*_*G*_, *M*). The standard convolution is calculated as:

$$G_{\text{k},l,n}=\sum_{i,j,m}K_{i,j,m,n}\cdot F_{k+i-1,l+j-1,m}$$ (2)

where, *D*_*F*_ × *D*_*F*_ represents the dimension of input feature map whose side length is *D*_*F*_; *D*_*G*_ × *D*_*G*_ represents the dimension of output feature map whose side length is *D*_*G*_; *D*_*K*_ × *D*_*K*_ is the product of the width and height of the convolution kernel; *M* and *N* represent the number of input and output channels respectively. Then the computational quantity Eq. of the standard convolution [Eq (3)] is:

$$C_{1}=D_{K}\cdot D_{K}\cdot N\cdot M\cdot D_{F}\cdot D_{F}$$ (3)

As depicted in Fig 7, the depth-separable convolution process encompasses two stages: deep convolution and 1×1 convolution. The calculation for deep convolution is defined by the following equation:

$${\hat{G}}_{\text{k},l,m}=\sum_{i,j}{\hat{K}}_{i,j,m}\cdot F_{k+i-1,l+j-1,m}$$ (4)

where, $\hat{G}$ and *F* are output feature maps and input feature maps respectively; Convolution kernel $\hat{K}$ with side length *D*_*K*_. k and *l* represent the *k* and *l* coordinates of the output feature map on the *m* channel; *i* and *j* are the convolution kernel coordinates of the *m* channel. The size of the deep convolution kernel is (*D*_*K*_, *D*_*K*_, 1), and there are *M* in total, which act on each input feature channel. The size of the output feature map is (*D*_*G*_, *D*_*G*_, *M*); The computational amount of deep convolution is derived from Eq (5):

$$C_{2}=D_{K}\cdot D_{K}\cdot M\cdot D_{F}\cdot D_{F}$$ (5)

The size of the point-wise convolution kernel is represented as (1, 1, *M*), and there are a total of *M* kernels, matching the number of channels in the output feature map. The output feature dimension is denoted as(*D*_*G*_, *D*_*G*_, *N*). The calculation for the point-wise convolution is as follows:

$$C_{3}=N\cdot M\cdot D_{F}\cdot D_{F}$$ (6)

Hence, the Eq (7) calculates the ratio of computational load between depth-separable convolution and conventional convolution.


$$\frac{W_{1}}{W_{2}}=\frac{M\cdot D_{K}^{2}\cdot D_{F}^{2}+M\cdot N\cdot D_{F}^{2}}{M\cdot N\cdot D_{K}^{2}\cdot D_{F}^{2}}=\frac{1}{N}+\frac{1}{D_{K}^{2}}$$ (7)

Building upon the two previously discussed convolution computation approaches, it can be deduced that depth-separable convolution facilitates the reduction of feature map dimensions through its utilization of convolutional filtering and feature fusion techniques. Consequently, the MobileNet network can be tailored for deployment on compact embedded devices, enabling the efficient classification and recognition of gesture images.

#### 3.3.2 Feature extraction based on multi-scale convolution

Traditional convolution employs a single filter for feature extraction, leading to a restricted receptive field that limits the network’s feature expression. This constraint is particularly noticeable in image classification tasks where various uncontrollable factors impact the resolution of gesture images. The use of a single convolution kernel with a fixed specification confines the range of feature learning within the network, resulting in weaker feature expression and diminished discrimination of image features. As a consequence, the network’s classification performance is compromised. Designing an effective model requires a balance between the number of parameters and network performance. To enhance recognition accuracy and the network’s adaptability, the most direct approach involves deepening the network structure. However, increasing network depth demands high-performance hardware resources for efficient network training. Failure to meet these hardware requirements may lead to excessively long training times, parameter proliferation, and potential issues such as gradient explosions.

**Fig 9:**
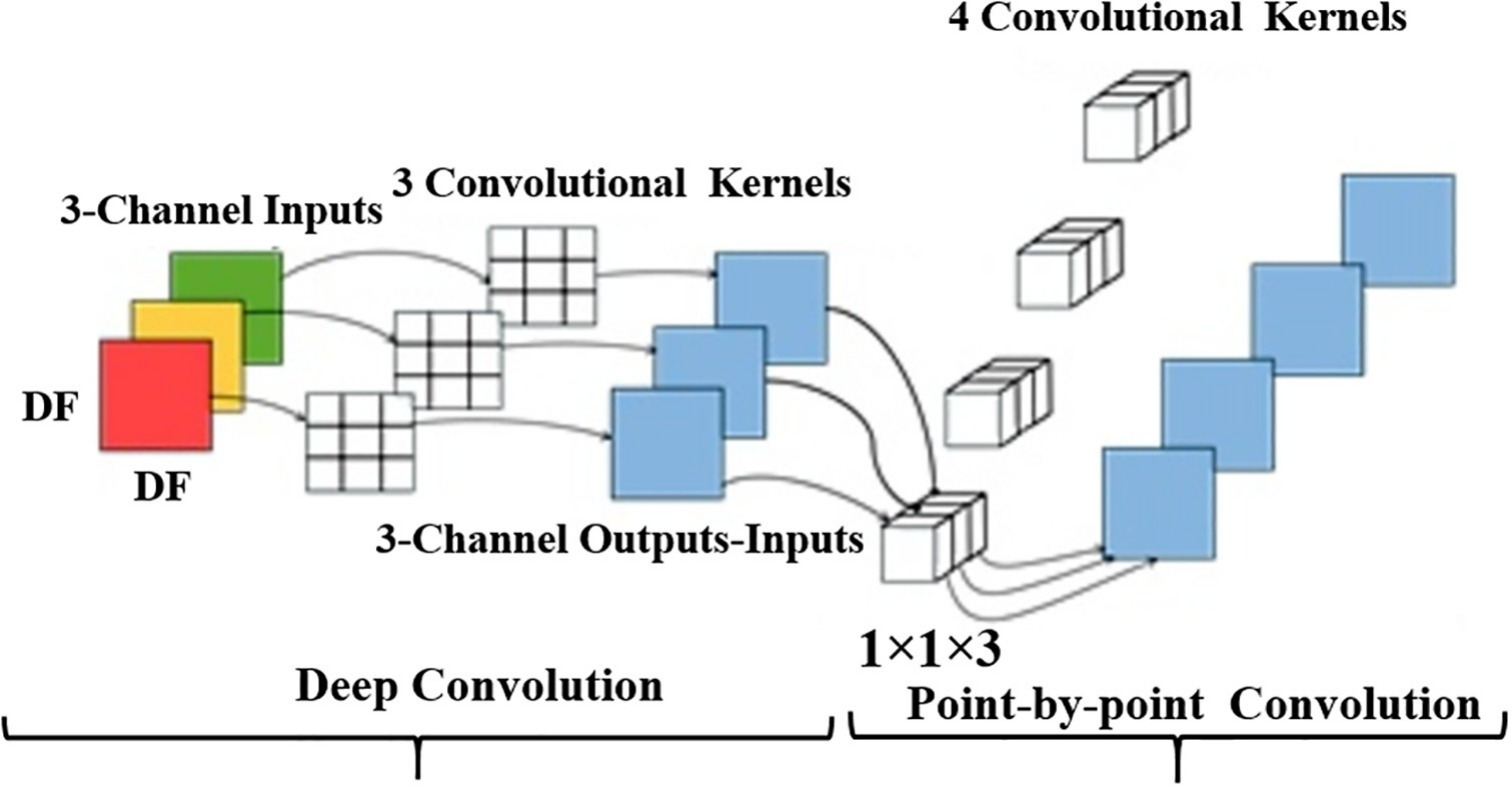
Multiscale convolution module.

To address these challenges, the concept of multi-scale feature extraction is introduced. Within the Multiscale Convolution modules (MS-Conv), smaller convolution kernels capture more localized feature information, while larger kernels emphasize abstract information. Through concatenating multi-scale convolutional kernels, diverse feature learning receptive fields are obtained, facilitating the extraction of feature information at different levels. This ensures that the output feature map effectively incorporates both detailed and abstract information from gesture images. Consequently, to devise a lightweight network model with high computational efficiency, a gesture recognition model based on the MS-MobileNet network is proposed, utilizing multi-scale convolution to replace the standard convolution in MobileNet. Fig 9 illustrates the multi-scale convolutional module.

The multi-scale convolution module uses convolution kernels with side lengths of 1×1, 3×3 and 5×5, and each feature extraction channel performs convolution operations on the input gesture images respectively. The multi-scale convolution operation is written as,

$$\left\{\begin{array}{l} F_{i}^{1\times 1}=f\left(\sum_{i=1}^{N}X_{j}*K_{ij}^{1\times 1}+b_{i}^{1\times 1}\right)\\ F_{i}^{3\times 3}=f\left(\sum_{i=1}^{N}X_{j}*K_{ij}^{3\times 3}+b_{i}^{3\times 3}\right)\\ F_{i}^{5\times 5}=f\left(\sum_{i=1}^{N}X_{j}*K_{ij}^{5\times 5}+b_{i}^{5\times 5}\right) \end{array}\right.$$ (8)

in Eq (8), *f*(.) is a nonlinear activation function; *N* is a subset of the input feature map; *X*_*j*_ is the *j* feature map of the input data layer; $K_{ij}^{1\times 1}$ is the 1×1 convolution kernel; $b_{i}^{1\times 1}$ is the offset term; $F_{i}^{1\times 1}$ is represented as the *i*-th output feature graph on the 1×1 convolution channel; $F_{i}^{3\times 3}$ and $F_{i}^{5\times 5}$ operate on the same principle as $F_{i}^{1\times 1}$.

Feature fusion operation is performed on the feature maps obtained from each channel of $F_{i}^{1\times 1}$, $F_{i}^{3\times 3}$ and $F_{i}^{5\times 5}$. The fused multi-scale feature maps can maximize the retention of key feature information of the input image and prevent the loss of local information. The Eq. for feature fusion is expressed as follows:

$$F_{i}=concat(F_{i}^{1\times 1}⊕F_{i}^{3\times 3}⊕F_{i}^{5\times 5})$$ (9)

in Eq (9), *F*_*i*_ is the output feature fusion graph; *concat* is the characteristic cascade operation function. ⊕ is the characteristic branch fusion operator.

During the feature mapping process, the configuration of branch channels plays a crucial role in determining the output feature dimension of multi-scale convolution. Employing a rapid splicing method for each branch channel facilitates the integration of multi-layer feature information, enhancing the network’s adaptability to various image resolutions. This approach also improves the learning and expressive capabilities of features. For the three branch channels *O*_*α*_, *Y*_*β*_, and *Z*_*ω*_ to be fused, the number of output channels after fusion is set to Sc, whose calculation Eq (10) is as follows:

$$S_{c}=O_{\alpha }+Y_{\beta }+Z_{\omega }$$ (10)

To address the issue where the input data distribution of each layer is influenced by the parameter adjustments of the preceding layer during training, a Batch Normalization layer (BN) is incorporated after each convolutional layer. This addition ensures that the mean and variance of the data conform to a standard normal distribution, thereby mitigating the complexity of network training. This not only accelerates the convergence speed of model learning but also guards against the problem of gradient disappearance. It is expressed as follows:

$$y_{i}=\gamma \frac{x_{i}-E}{\sqrt{V+\varepsilon }}+\varphi =BN_{\gamma ,\varphi }\left(x_{i}\right)$$ (11)

in Eq (11), *ε* is a positive number approaching 0, which is set to satisfy the fractional equation; *γ* and *φ* are the reconstruction parameters learned during training. *E* is the mean value of each batch sample *x*; *V* is the variance.

Integrating multi-scale convolution enhances the network’s architecture, extending the scope of feature extraction, strengthening the network’s ability to discern features, stabilizing its adaptability to multi-scale features, and contributing to the optimization of model parameters and computational requirements. Eq (12) represents the number of parameters for a multi-scale convolutional module, while Eq (13) defines the calculation quantity as follows:

$$P=\sum_{m=1}^{n}K_{v}^{2}c_{m-1}c_{m}$$ (12)

$$Q=\sum_{m=1}^{n}H_{m}W_{m}K_{v}^{2}c_{m-1}c_{m}$$ (13)

in Eqs (12) and (13), *n* is the convolution layer; *K*_*v*_ is the filter; *c*_*m*−1_ and *c*_*m*_ represent the number of input and output channels of the *m*-th convolutional layer. *H*_*m*_ and *W*_*m*_ represent the height and width of input features of the *m*-th convolutional layer.

#### 3.3.3 Improvement of activation function

**Fig 10:**
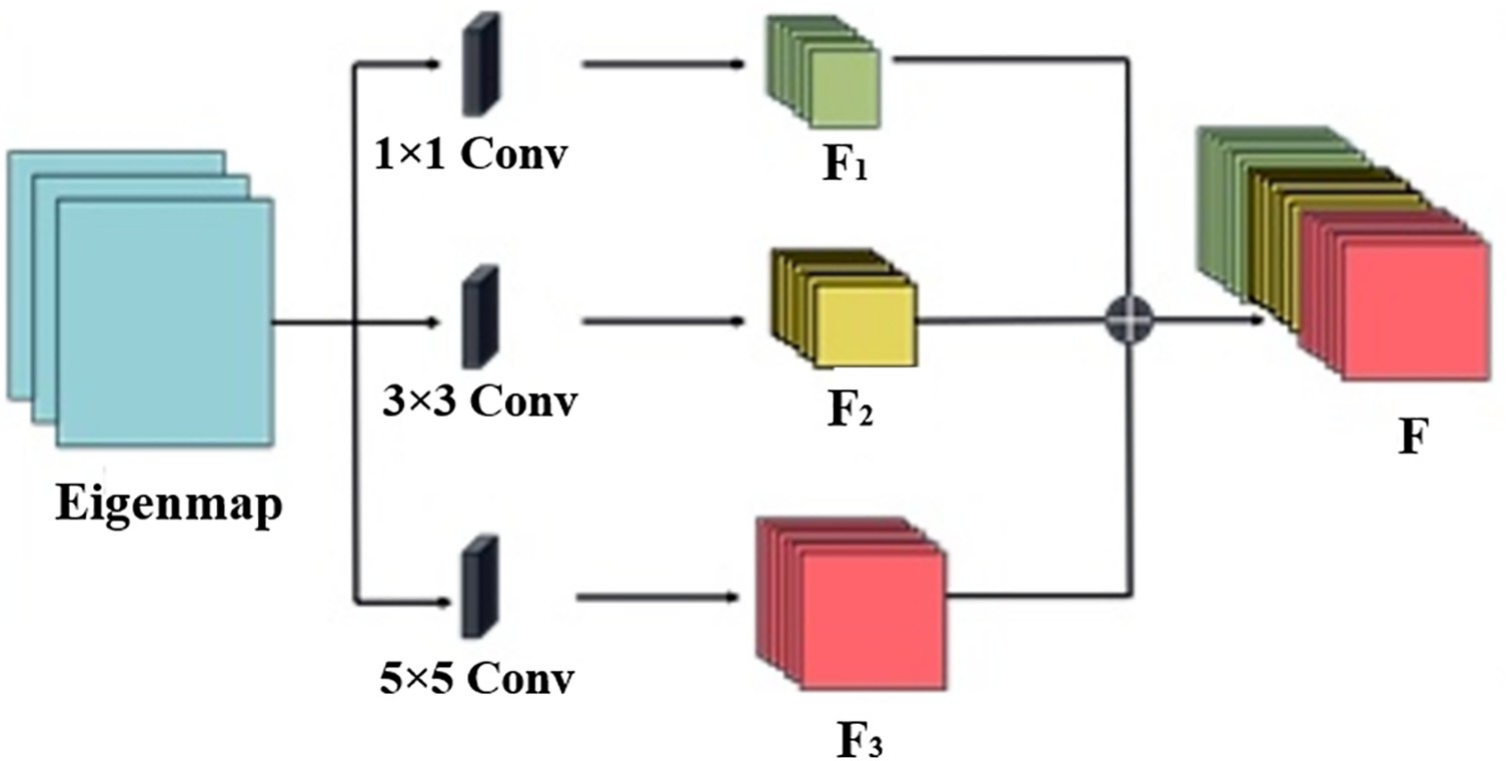
Comparison of ReLU and ELU activation functions.

Selecting the appropriate activation function is critical for introducing nonlinearity into neural networks and has a substantial impact on the performance and accuracy of gesture image recognition. In the field of image processing, ReLU, PReLU, Tanh, and Sigmoid are prominent activation functions. ReLU can converge faster in the training process of neural networks, and can effectively solve the problem of gradient disappearance, and after the introduction of sparsity, it helps to reduce the complexity of the model and the number of parameters. ReLU is widely used in deep neural networks, especially in convolutional neural networks (CNNS), where it is favored for its simplicity and high efficiency. PReLU is an improved form of ReLU, which allows neurons to have different activation thresholds and slopes, improving the flexibility and performance of the model. However, compared with ReLU, PReLU adds additional parameters, which will increase the complexity of the model. The Tanh output ranges from -1 to 1 and has a zero-centered nature, but when the input value is large or small, the gradient will be close to zero, which may cause the gradient disappearance problem. The Sigmoid output boundary is between 0 and 1, which is easy to interpret and translate into probabilities. However, when the input value is large or small, the gradient will also appear close to zero, resulting in slow or stagnant training process. Among these, the ReLU activation function stands out due to its high computational efficiency and its ability to address the vanishing gradient problem. This can be visually observed in Fig 10.

The ReLU mathematical Eq (14) is:

$$f(x)=\left\{\begin{array}{l} x,x\geq 0\\ 0,x<0 \end{array}\right.$$ (14)

It can be seen from Eq (14) that the function is a piecewise function, which does not meet the requirement of full interregion derivability, and can use the property of nonlinear function to deal with the complex relationship in the data. When the input value *x* is greater than 0, *f*(*x*) is equal to the input value, there is no gradient saturation, and the slope of *f*(*x*) is 1, and the gradient remains proportional to the node activation, which can effectively alleviate the problem of gradient disappearance. When the input value *x* is negative or 0, the derivative of *f*(*x*) is always 0, the activation effect on the data disappears, and the phenomenon of neuron "necrosis" occurs, so that the weight cannot be updated. Moreover, this process is irreversible, which affects the diversity of data and leads to non-convergence of calculation results.

**Fig 11:**
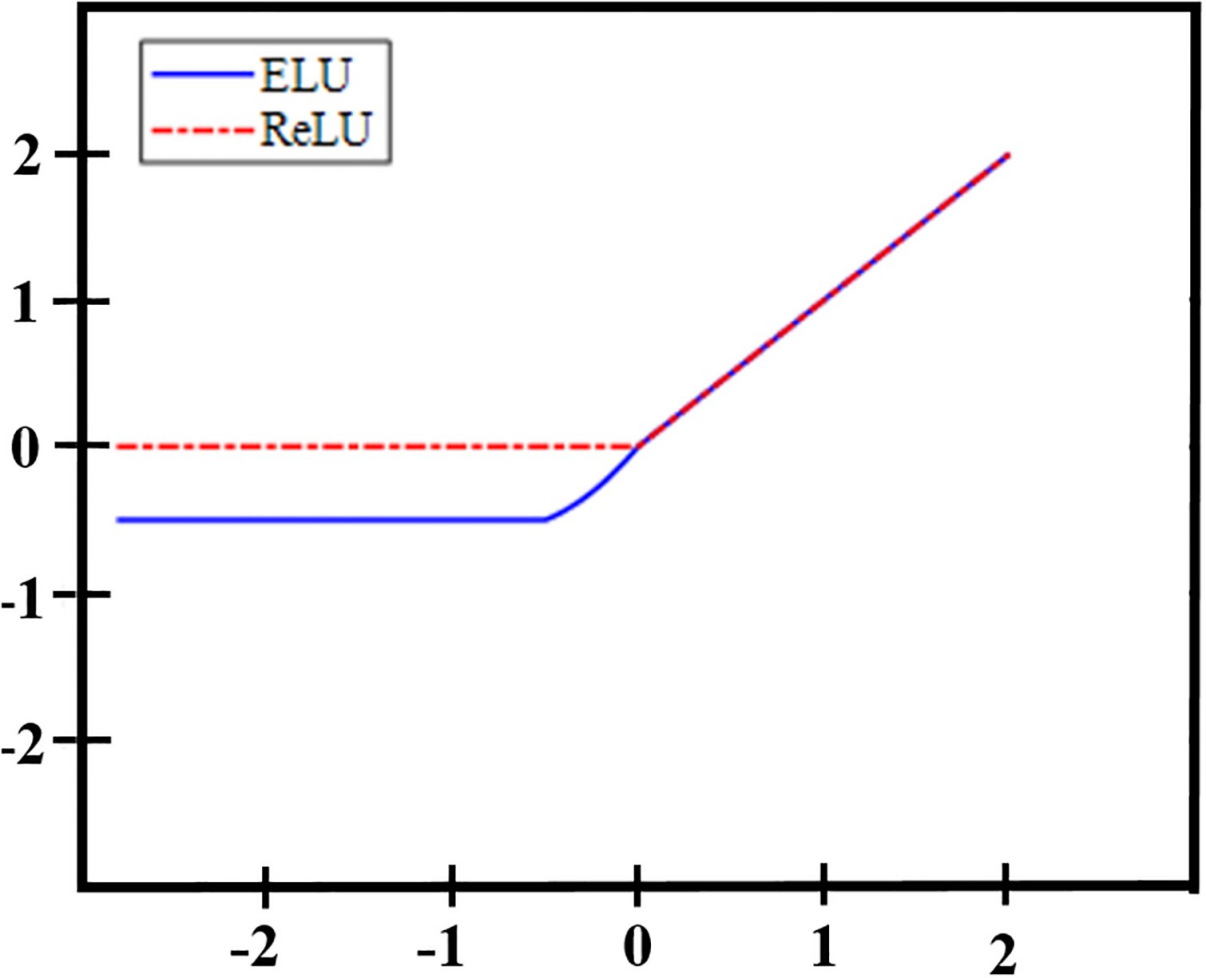
MS-MobileNet structure diagram.

Taking into account the generalization ability of the MS-MobileNet network in image recognition tasks and the limitations of the ReLU function, replacing the ReLU function with the ELU function in deep separable convolution can preserve the advantages of the ReLU function while addressing its shortcomings. The ELU image is depicted in Fig 11 and is mathematically defined as H represented by Eq (15), with its derivative outlined in Eq (16):

$$f(x)=\left\{\begin{array}{c} x,x>0\\ a\left(e^{x}-1\right),x\leq 0 \end{array}\right.$$ (15)

$$f^{\prime }(x)=\left\{\begin{array}{c} 1,x>0\\ f^{\prime }(x)+\alpha ,x\leq 0 \end{array}\right.$$ (16)

where *α* is an adjustable parameter, usually set to a constant between 0.1 and 0.3. As can be seen from Figs 3–6, 11 and the Eq (16), compared with ReLU function, when the value of x falls on the positive semi-axis, it remains unchanged; However, when *x* is in the negative semi-axis, the gradient is no longer 0, and the characteristics of the negative region are retained, so that the adaptability of the ELU function to the input value is enhanced, the convergence rate is accelerated, and the problems such as gradient disappearance or explosion can be alleviated.

## 4. Experiment and result analysis

### 4.1 Experimental setup

**(1) Experimental environment.** In this investigation, experiments were conducted using the MS-MobileNet network on a 64-bit Windows 10 operating system, leveraging an NVIDIA GeForce RTX 2060 SUPER graphics card with 8GB of video memory. Python 3.8 served as the programming language, and the Tensorflow 1.13 framework, along with Tensorflow 1.13 developer tools, was employed. The image dimensions were configured, the initial learning rate was set to 0.001, a batch size of 32 was chosen, and the Adam optimizer was utilized in conjunction with the cross-entropy loss function. Classification was executed using Softmax.
**(2) Evaluation indicators.** Various evaluation criteria have been employed to quantitatively assess the model’s performance objectively. Throughout the experiment, we use accuracy rate, recall rate, and accuracy as benchmark indicators to evaluate the precision of the gesture recognition algorithm. Additionally, the number of floating-point operations and parameters are utilized as reference metrics for assessing the model’s operational complexity.
**(3) Experimental data set.** Demonstrating improved recognition capabilities and emphasizing its practical significance, the implementation of a gesture recognition algorithm in real-world scenarios requires overcoming substantial interference from various factors. To verify the validity of the model, this study used multiple gesture datasets, including ASL-M, NUS-II, and NVGesture. NUS-II is a dataset of hand posture created by the National University of Singapore. The gestures in this dataset were presented by 40 subjects against a variety of complex backgrounds, and it contains image data of multiple gestures against complex backgrounds. The data set provides rich experimental data and verification platform for gesture recognition research, which is helpful to promote the development and application of gesture recognition technology. Studies have shown that the static gesture recognition algorithm based on the combination of YCbCr color space and convolutional neural network has a recognition accuracy of 98.32% on the NUS-II gesture data set. NVGesture is a dynamic gesture recognition database focused on contactless driver control. It contains 1532 dynamic gestures, divided into 25 categories. The data set included 1050 samples for training and 482 samples for testing. To validate the model’s efficacy, multiple gesture datasets, including ASL-M, NUS-II, and Creative Vesenz3D, were utilized in this study. To fortify the model’s resilience, the dataset was further enriched through experimental techniques such as random clipping and translation adjustments. These augmentations enhance the model’s performance and act as a safeguard against overfitting. Some parts of face recognition datasets were provided in S1 Table (see Supporting information).

### 4.2 Validity verification of skin color detection

**Fig 12:**
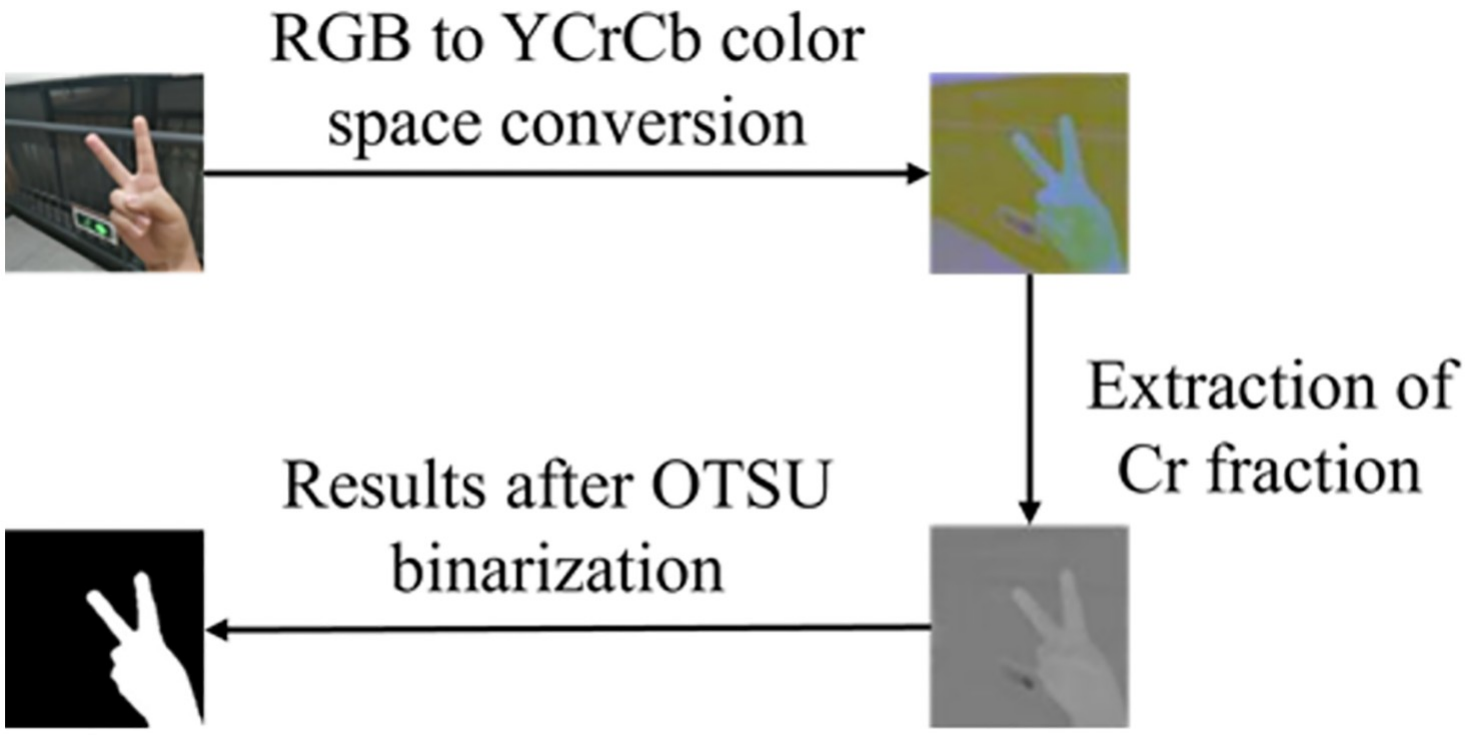
Skin color extraction process result chart.

**Fig 13:**
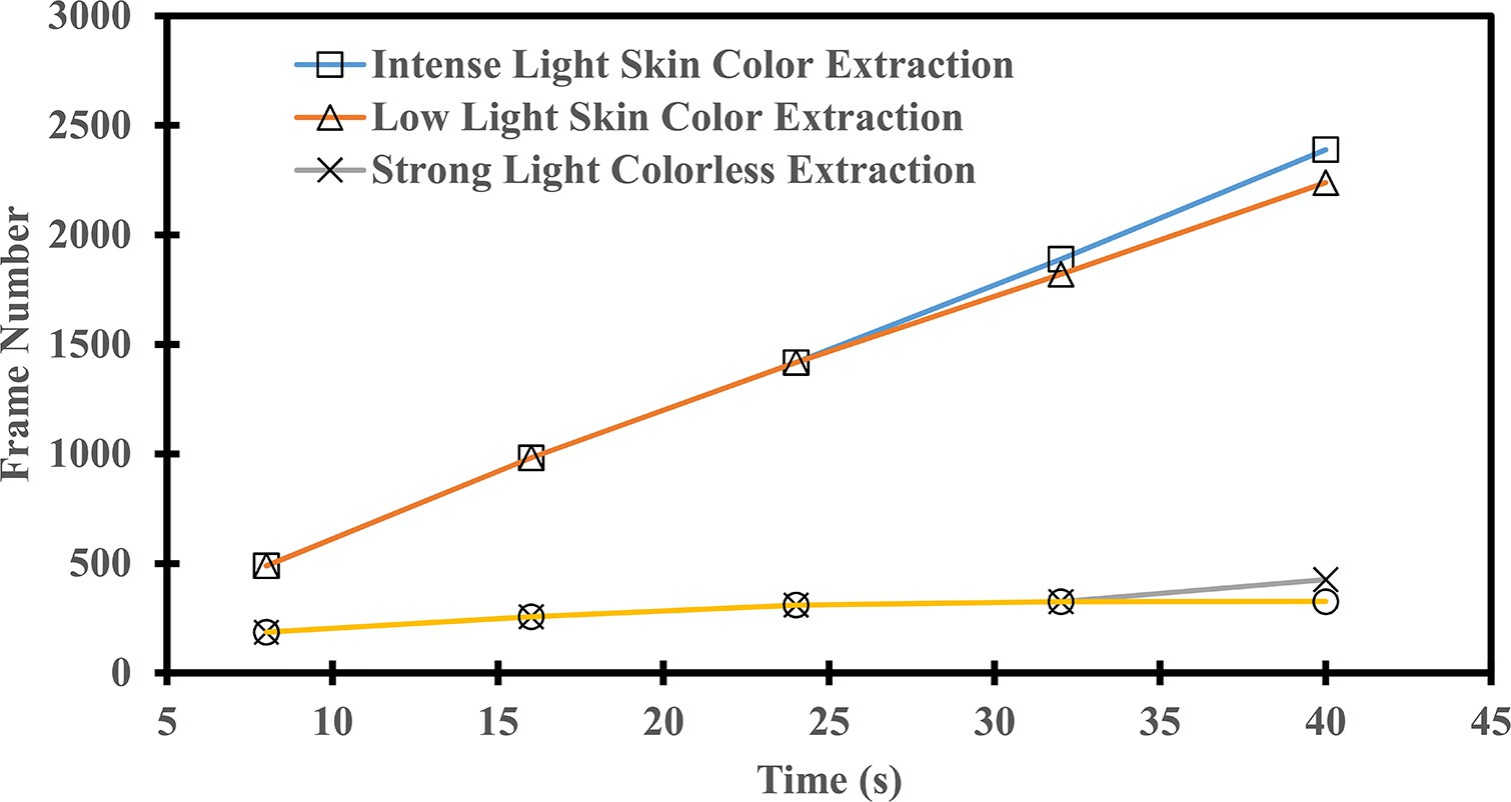
Skin color extraction performance comparison chart.

The rendering effect varies depending on the image quality in the RGB color space, primarily influenced by changes in light intensity. In low-light conditions, the sensor captures diminished light signals, resulting in relatively higher noise levels and a diminished image recognition rate. Conversely, bright light conditions yield the opposite effect. Verification is conducted based on two factors: skin color detection and light intensity. Fig 12 illustrates the hand’s skin color extraction process, while Fig 13 quantitatively evaluates the performance difference in skin color segmentation. Datasets of Fig 13 was provided in S2 Table. As depicted in Fig 13, during a specific time frame, frame extraction is more effective in well-lit environments compared to low-light settings. Given the same conditions, images with skin color detection facilitate the capture of more frames, contributing to a relatively stable recognition outcome. The efficacy of the skin color segmentation algorithm employed in this experiment is validated, offering valuable insights for optimizing the network structure and enhancing the algorithm’s generalization capabilities.

### 4.3 Comparative experiment

To assess the performance effects of the proposed model in various scenarios, experiments were conducted on publicly available datasets NUS-II [27] and Creative Senz3D [29]. Table 2 presents a comparison of recognition performance on these two commonly used datasets. Programming codes of CNN model was provided in the S1 File.

**Table 2:** Performance comparison on NUS-II and creative Senz3D datasets.

Model Name	ANUS-II/%	AC-S/%
Algorithm of this paper	92.55	88.41
MobileNetV1	87.23	85.37
MobileNetV2	88.46	85.30
SqueezeNet	88.27	86.03
EfficientNet	90.63	87.34
AlexNet	89.31	86.29

The outcomes unequivocally indicate that our proposed model achieves an accuracy of 92.55% on the NUS-II dataset and 88.41% on the Creative Senz3D dataset. These scores outperform those attained by the other five models. Although the enhancement in performance compared to MobileNetV1 is notably observable, it doesn’t exhibit a significant contrast compared to the remaining models. This implies that the model’s perceptual capability may be compromised in the presence of insufficient data, ultimately diminishing its effectiveness in gesture classification.

In this study, we assessed the performance of the gesture recognition algorithm utilizing the ASL-M dataset. Table 3 provides details on nine sets of comparative experiments, involving models such as AlexNet, EfficientNet, and MobileNetV1.

**Table 3:** Performance comparison of different models on ASL-M dataset.

Model Name	Accuracy/%	FLOPs/MB	Params/MB
AlexNet	95.21	714	61.10
GoogleNet	90.26	1504	6.62
ResNet50	94.32	4111	25.56
MobileNetV1	95.68	575	4.21
MobileNetV2	95.10	314	3.50
MobileNetV3	93.47	312	3.52
ShuffleNet	92.39	148	2.28
SqueezeNet	95.06	823	1.25
EfficientNet	96.37	399	5.30
Algorithm of this paper	98.26	580	4.23

The recognition accuracy has seen a slight improvement, reaching a rate of 98.26%, which is 2.58% higher than that of MobileNetV1, as indicated in the table. However, the number of parameters and computational workload does not exhibit a significant deviation from the standard MobileNetV1 model. In comparison to MobileNetV2 and MobileNetV3, which have an equal parameter count, our model’s computational size has expanded by more than 270MB, resulting in recognition accuracy increments of 3.16% and 4.79%, respectively. These findings underscore the effectiveness of the multi-scale convolutional module. To enhance the precision of gesture recognition, the model captures features across various scales, broadening the scope of feature learning within the network and bolstering the network’s perceptual capabilities. It achieves this by incorporating the ELU activation function to extract critical information more comprehensively. In contrast, ShuffleNet, SqueezeNet, and EfficientNet, three lightweight networks that can be compared with the model presented in this paper, exhibit certain advantages but fall short in terms of accuracy, making our model the most promising choice. The model’s adaptability remains largely unaltered by the computational load or the quantity of parameters, even though these aspects are not particularly pronounced. This model operates with enhanced efficiency. In comparison to the conventional AlexNet, GoogleNet, and ResNet50 models, the proposed model boasts reduced parameter counts and demands less computation. Surprisingly, it exhibits varying degrees of improved accuracy, with increments of 3.05%, 8.0%, and 3.94%, correspondingly. This observation underscores the notable potential of deep separable convolution in substantially curtailing network parameters, conserving computational resources, mitigating feature information loss stemming from pooling procedures, and elevating recognition accuracy.

The algorithm, as outlined in this study, has been implemented on mobile devices for test analyses, aligning with the requirements of gesture application scenarios. The results of these tests are presented in Table 3.

Table 4 illustrates that, in comparison to the MobileNetV1 model, our proposed model demonstrates faster convergence, shorter training durations, and quicker recognition. It achieves an impressive single recognition time of just 0.115s, representing a 0.017s improvement over MobileNetV1. Furthermore, its speed outperforms that of other comprehensive models. The network’s real-time performance is exceptional, effectively meeting the requirements for real-time gesture recognition on mobile devices.

**Table 4:** Comparison of recognition efficiency of models.

Item	Single iteration speed /s	Identification time /s
MobileNetV1	652	0.132
Algorithm in this paper	561	0.115

**Fig 14:**
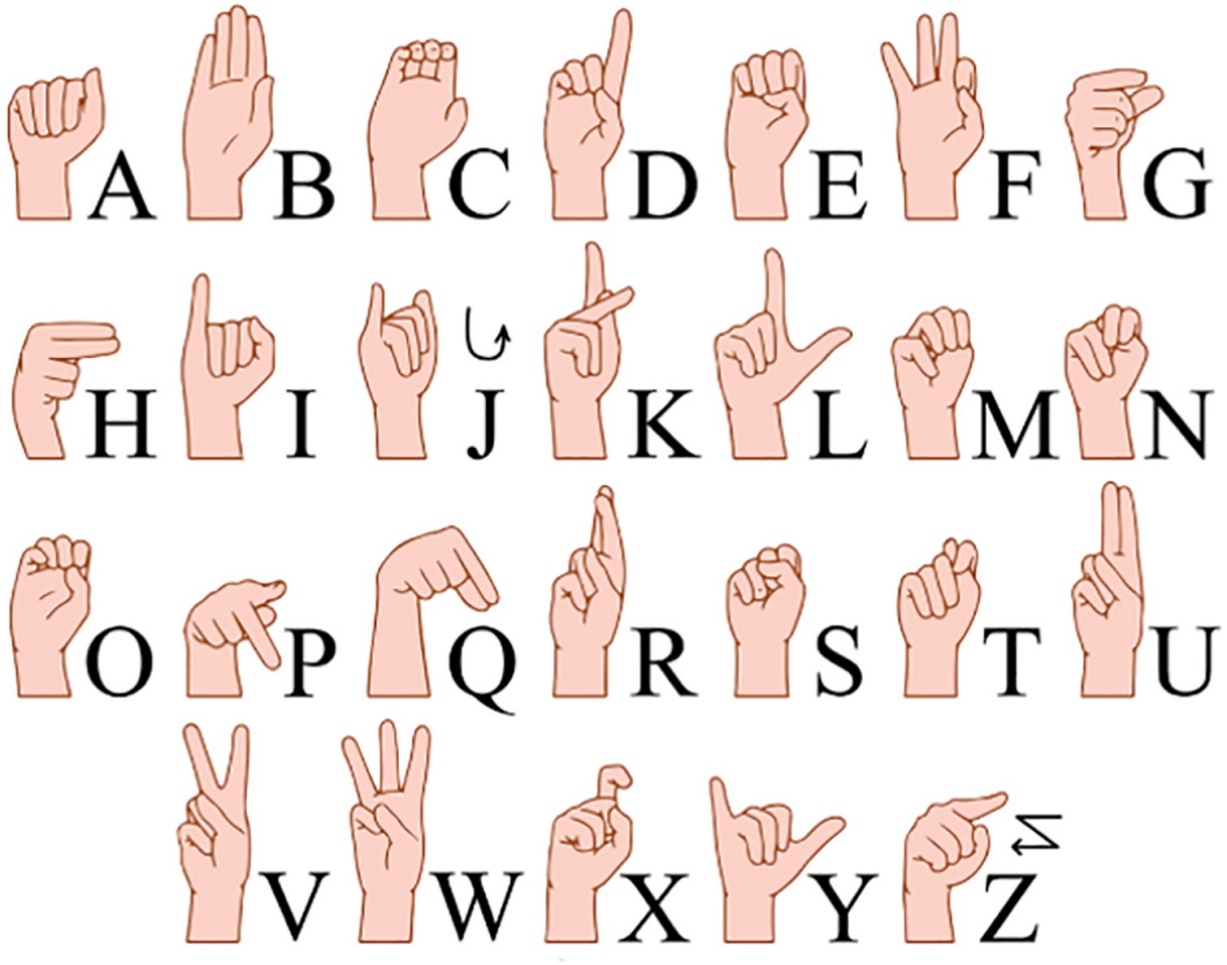
Confusion matrix for letter gestures [30].

For a thorough investigation into the generalization capability of gesture recognition, we shift our focus to Table 5, which displays the test results for 24 distinct letter gestures. The overall performance of this model is notably commendable. However, when it comes to letter gestures M and N, distinguished by significant self-similarity, the recognition proficiency is less than optimal. This underscores the critical necessity of improving the model’s generalization aptitude in such scenarios. The confusion matrix is shown in Fig 14.

**Table 5:** Alphabet gesture recognition performance metrics for different categories.

Sign	Precision	Recall	F1-score	Sign	Precision	Recall	F1-score
A	0.99	0.99	1.00	N	0.95	0.97	0.96
B	1.00	1.00	1.00	O	1.00	1.00	0.96
C	1.00	0.99	0.99	P	0.96	0.98	0.98
D	1.00	0.98	0.99	Q	0.98	0.99	0.98
E	0.97	0.97	0.98	R	0.98	1.00	0.99
F	1.00	1.00	1.00	S	1.00	1.00	1.00
G	0.97	0.99	0.99	T	0.98	0.98	0.98
H	0.96	0.95	0.95	U	1.00	0.99	1.00
I	0.97	0.99	0.98	V	1.00	1.00	1.00
K	0.96	0.94	0.96	W	1.00	1.00	1.00
L	1.00	1.00	1.00	X	0.97	0.98	0.98
M	0.94	0.92	0.96	Y	1.00	1.00	1.00

### 4.4 Ablation experiment

In our study, we designed four distinct test sets, incorporating both individual and combined enhancements. These tests were Eq.ted to assess the impact of the multiscale convolution module and the ELU activation function on the effectiveness of our proposed algorithm. The goal was to comprehensively investigate how different components influenced the overall system performance. Firstly, MobileNet model is determined as a baseline test set, and only multi-scale convolution module is added to the baseline model to observe its impact on system performance. Based on the baseline model, only the activation function was replaced with ELU to evaluate its improvement in system performance. The multi-scale convolution module and ELU activation function are added to the baseline model to evaluate the comprehensive effect of the combination on the system performance. Then, MobileNet+ ELU, MobileNet+MS-Conv and MobileNet+MS-Conv+ELU models are generated on the basis of MobileNet model. The experimental steps include data preparation, model modification training and evaluation, and then compare and analyze each model.

The results of these experiments are summarized in Table 6. The analysis of the table shows that the MobileNet model obtained an accuracy of 95.68% and a recall of 94.03%. In the second set of experiments, we exclusively replaced the ReLU activation function with ELU, leading to a marginal improvement in recognition performance. In the third set of experiments, we constructed the MobileNet+MS-Conv model by combining the multi-scale convolution technique with the Eq.tion used for multi-channel feature extraction. This integration significantly improved the accuracy of the original MobileNet model by 1.45%. Turning our attention to the fourth set of experiments, the joint enhancement study outlined in this paper, the MobileNet+MS-Conv+ELU model, it can be seen through the data that the models in this paper show a large improvement compared to both models in the previous set. Specifically, the accuracy rate soared by 2.6%, while the recall rate increased dramatically by 4.7%. The results obtained from the ablation experiments indicate that the various branching modules do contribute positively to the recognition accuracy. However, it is worth noting that the combined improvement approach produced the most impressive results in terms of enhancing the effectiveness of gesture recognition.

**Table 6:** Ablation experiment.

Model name	Accuracy/%	Recall/%
MobileNet	95.68	94.03
MobileNet+ ELU	96.30	96.54
MobileNet+MS-Conv	97.13	97.82
MobileNet+MS-Conv+ELU	98.26	98.69

## 5. Conclusion

This study employs a skin color detection method to accurately locate the gesture region, addressing the challenges of the MobileNet network in terms of extracting gesture features and reducing error rates in identification. Subsequently, a multi-scale convolutional module is developed to extract deep and shallow features simultaneously using convolutional kernels of different sizes. This approach aims to express gesture features more efficiently and enhance the network’s capability for feature extraction. Finally, the activation functions of ReLU and ELU are compared and studied. The ELU function is chosen as the activation function because it focuses more on negative feature information, accelerating the network’s training process and improving gesture recognition accuracy.

In order to further improve the practicality of gesture recognition, non-hand information assistance such as facial expressions and body movements will be combined in the future to assist sign language recognition, reproduce actual sign language communication scenes, and promote intelligent sign language recognition. Establish a fully detailed database of sign language words and continuous sign language, and implement sign language research as soon as possible to serve practical life.

## Supporting information

S1 Table Face recognition datasets. (XLSX)

S2 Table Frame number variation for skin color extraction versus time. (XLSX)

S1 File Convolutional neural network. (RAR)
